# The shifting role of the cerebellum in executive, emotional and social processing across the lifespan

**DOI:** 10.1186/s12993-022-00193-5

**Published:** 2022-04-28

**Authors:** Pierre-Aurélien Beuriat, Irene Cristofori, Barry Gordon, Jordan Grafman

**Affiliations:** 1 Cognitive Neuroscience Laboratory, Brain Injury Research, Shirley Ryan AbilityLab, Chicago, IL USA; 2 Feinberg School of Medicine, Northwestern University, Chicago, IL USA; 3 Department of Pediatric Neurosurgery, Hôpital Femme Mère Enfant, Hospices Civils de Lyon, Lyon, France; 4 Rockfeller School of Medicine, Claude Bernard University, Lyon, France; 5 Institute of Cognitive, Neuroscience Marc Jeannerod, CNRS/UMR 5229, 69500 Bron, France; 6 Université Claude Bernard, Lyon 1, 69100 Villeurbanne, France; 7 Department of Neurology, Johns Hopkins University School of Medicine, Baltimore, MD USA; 8 Department of Cognitive Science, Johns Hopkins University, Baltimore, MD USA; 9 Departments of Neurology, Psychiatry and Cognitive Neurology & Alzheimer’s Disease, Feinberg School of Medicine, Northwestern University, Chicago, IL USA

**Keywords:** Cerebellum, Executive functions, Emotion, Social behaviors, Children, Adults

## Abstract

The cerebellum’s anatomical and functional organization and network interactions between the cerebellum and the cerebral cortex and subcortical structures are dynamic across the lifespan. Executive, emotional and social (EES) functions have likewise evolved during human development from contributing to primitive behaviors during infancy and childhood to being able to modulate complex actions in adults. In this review, we address how the importance of the cerebellum in the processing of EES functions might change across development. This evolution is driven by the macroscopic and microscopic modifications of the cerebellum that are occurring during development including its increasing connectivity with distant supra-tentorial cortical and sub-cortical regions. As a result of anatomical and functional changes, neuroimaging and clinical data indicate that the importance of the role of the cerebellum in human EES-related networks shifts from being crucial in newborns and young children to being only supportive later in life. In early life, given the immaturity of cortically mediated EES functions, EES functions and motor control and perception are more closely interrelated. At that time, the cerebellum due to its important role in motor control and sequencing makes EES functions more reliant on these computational properties that compute spatial distance, motor intent, and assist in the execution of sequences of behavior related to their developing EES expression. As the cortical brain matures, EES functions and decisions become less dependent upon these aspects of motor behavior and more dependent upon high-order cognitive and social conceptual processes. At that time, the cerebellum assumes a supportive role in these EES-related behaviors by computing their motor and sequential features. We suspect that this evolving role of the cerebellum has complicated the interpretation of its contribution to EES computational demands.

## Introduction

The cerebellum has been assigned many crucial roles beginning with motor control but more recently it has been deemed crucial for higher-order cognitive, emotional, and even social processing [54]. However, the cerebellum’s organization and the interactions between the cerebellum and the cerebral structures are dynamic across the lifespan and Executive, emotional and social (EES) functions have likewise evolved during development from contributing to primitive behaviors during our early life to being able to direct complex actions as an adult. This parallel development indicates that integration of different cortical regions into varied functional networks is a dynamic process. These networks also rely upon subcortical structures important for motivation and reward, valuation, and sensorimotor filtering, processes which also evolved from childhood to adulthood. Therefore, the functional responsibility of each node in the brain network that support EES functions might have a different weighting across development. This might be also true for the cerebellum,however, no such hypothesis has previously been explicated.

In this review, we will suggest that the role of the cerebellum in human EES-related networks shifts from being crucial in newborns and young children to being only supportive later in life. Here, we propose, based on evidence from both the child and adult literatures, a dynamic framework for this shifting importance of the cerebellum in executive, emotional and social processing across the lifespan.

## From sensorimotor control to cognition

Children have to learn and then practice skilled movements and behavior in order to perform them without conscious attention whereas in adults, cognitive and motor behavior are coordinated, often unconsciously, and often become automatically expressed ensuring efficient performance.

EES behaviors that are acquired and eventually executed automatically are essential in real-life, otherwise, EES actions would impose a significant cognitive load. These automatic EES behaviors are not always sufficient when slower high-order cognitive regulation and adaptation is mandatory. Children and adults are often engaged in EES behaviors that elicit automatic or higher-order control. Sensorimotor-based perception and agency and high-order control are two distinct means of EES behavioral control that rely upon complex anatomical and functional networks that evolve during human development.

## The EES cerebellum in adults

### Evidence of the role of the cerebellum in EES functions from healthy volunteer and patient studies

#### Studies in healthy volunteers

**Fig. 1:**
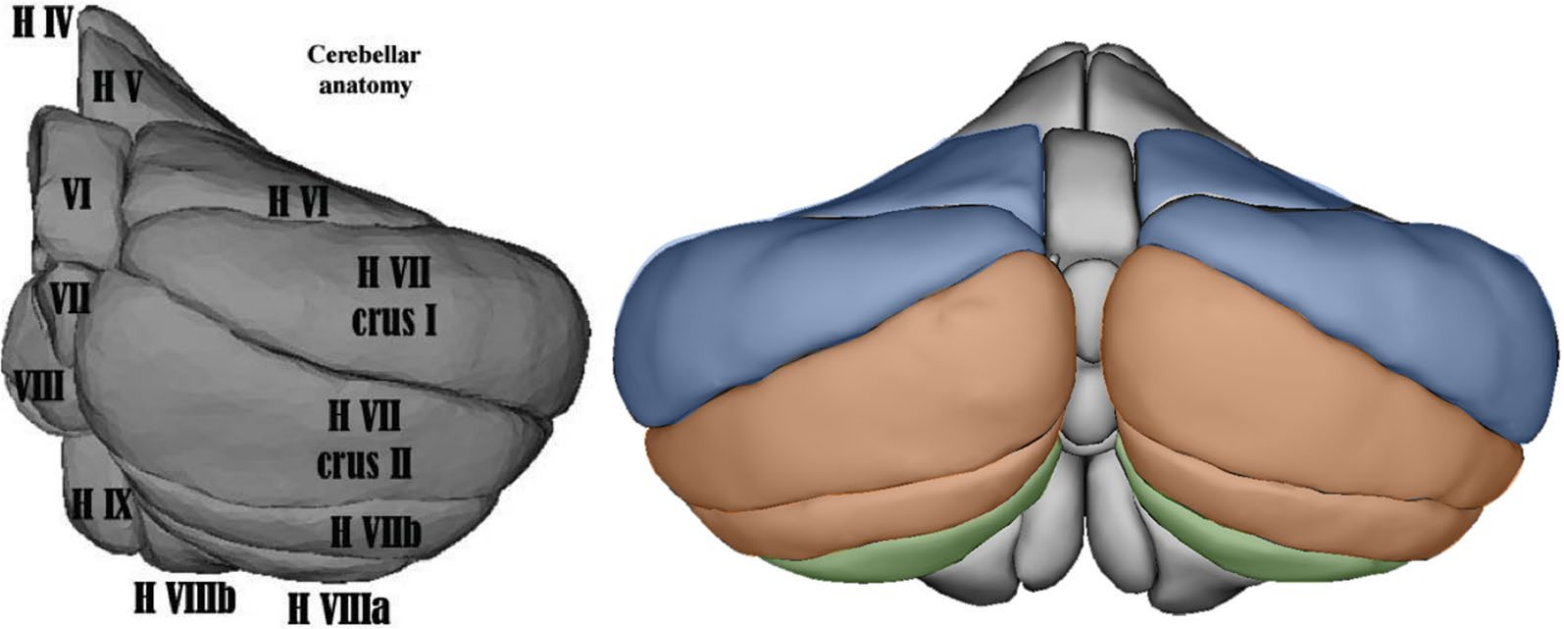
Right: anatomical representation of the cerebellum. Left: repartition of function within the cerebellum (blue: emotion, working memory and exécutive function, orange: working memory and exécutive function, green: working memory and emotion)

In adults, multiple task-based imaging studies report healthy volunteer cerebellar activation across a wide range of tasks, depending on task demands (for review see [57]. EES task related cerebellar activation sites are located as follows: working memory activates lobules VI, VII, and VIIIA, bilaterally; Executive functions (EF activates lobules VI and VII (VIIB, Crus I, Crus II, bilaterally and emotional processing activates lobules VI, Crus I, and VIIA, bilaterally and in the midline (Fig. 1). As for social cognitive tasks, multiple regions were reported to be involved with stronger evidence for functional activation in the right crus I and lobule IX [63]. It is worth noting here that motor activation sites were different as most of them were located in the anterior part of the cerebellum (lobule I–V) except when motor tasks were more complex (for a review see [57]). For example, when a motor task involved planning or sequencing, lobules VI and VII are activated, sometimes, in addition to the “cerebellar motor regions” [52].

#### Studies in cerebellar patients

Despite the enthusiastic attribution of cognitive and emotional functions to the cerebellum, there are still disputes regarding the exact role of the cerebellum in performing EES tasks [18]. In adult studies, neuropsychological task impairment has been reported after cerebellar damage in almost every high-order function including EF (cognitive flexibility, speed of processing, planning, reasoning, working memory, inhibitory control, problem solving) (for a review, see [58]) and several emotion processes (emotional perception, emotional recognition, emotional processing, emotional learning) (for a review, see [2]). Conversely, there are also many authors that reported very mild to no cognitive dysfunction in adult patients with cerebellar lesions [20, 22, 39, 40, 48] while others reported deficits on certain EF tests in cerebellar patients compared to controls, but often the cerebellar patients’ performance was still typically within the normal range [33, 47].

Lastly, to our knowledge, no perfusion imaging studies showing distant cortical hypoperfusion following cerebellar damage have been reported in adult patients.

#### EES anatomy and network

**Fig. 2:**
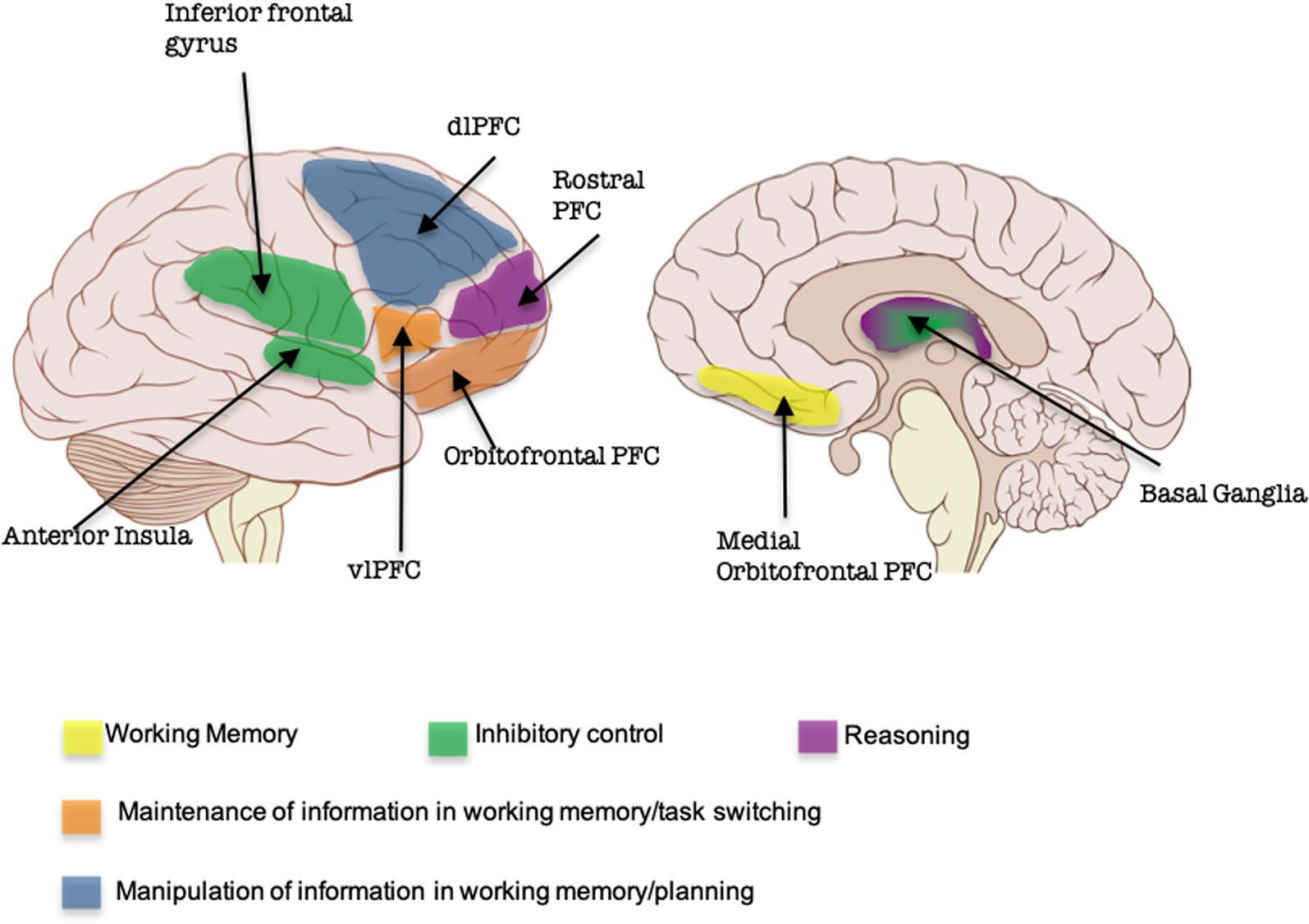
Cortical brain area associated with high order functions based on lesions and/or neuroimaging studies. Adapted from [10]

Overlapping brain regions support EES functions (Fig. 2). EF are dependent upon the prefrontal cortex (PFC). The PFC has multiple input and output projections with cortical and subcortical structures including the cerebellum. Input projections are from the parietal and temporal cortex, hippocampus, cingulate cortex, substantia nigra and thalamus. Output projections are to the thalamic medial dorsal nuclei as well as to the amygdala, the septal nuclei, the basal ganglia, and the hypothalamus. Thus, the PFC is one of the critical cortical regions that participate in extended EES neural networks. Yet the PFC doesn’t fully mature until the third decade of life.

The anatomical networks that are responsible for emotional processing are also extensive as the different manifestations of emotion (behavioral, autonomic, or cognitive) are closely intermingled with other brain functions such as motor skills, cognition, and sensory processes. The core of the system that processes emotion includes the PFC, the hippocampus, the amygdala, the hypothalamus, the pituitary gland and the insula. Others subcortical regions such as the basal ganglia (thalamus, striatum, globus pallidus, subthalamic and accumbens nuclei) are connected to these regions by circuits known as Cortico-Striato-Thalamo-Cortical loops [34]. These latter midline structures that extend to the ventromedial PFC may “color” the emotion system with valuation scaling leading to an interaction between emotion, reward, desire, and agency.

Social functions are also supported by an extended brain network, including dorsomedial PFC, premotor cortex, intraparietal sulcus, temporoparietal junction, posterior cingulate cortex, and ventro medial PFC, dorso lateral PFC, Orbito Frontal Cortex, and the anterior temporal lobe [21].

These EES functions closely interact, therefore several overlapping brain networks involve these functions. All these cortical and subcortical supra tentorial regions are known to be anatomically and functionally connected either directly or indirectly to the cerebellum forming a larger distributed network concerned with EES functions.

#### EES brain networks with cerebellum membership

EES networks including the cerebellum can be considered both functionally and anatomically as “closed-loop” systems. These integrated systems have been continuously evolving throughout anthropoid evolution as species acquired complex skills. Almost every cortical area has a reciprocal, segregated connection with the cerebellum although these connections may traverse polysynaptic circuits [58]. The thalamus is a major relay from/to the cerebellum to/from the cortex. In the cerebellum, the deep cerebellar nuclei, especially, but not exclusively, the dentate nuclei (DN), are the gateway and the entry nodes of the loop from/to the cerebellum. In adult humans, distinct cortico-cerebellar circuits have also been mapped from/to the cerebellum from/to the motor and the PFC [30]. However, compared to non-human primates, in humans the connection of the posterior cerebellum only with motor cortex and of the anterior cerebellum only with PFC is not respected. Lobule VIIIb is additionally connected to the motor cortex and lobule VI to the PFC [30]. These cortico-cerebellar loops rely on white matters tracts between the DN and the prefrontal cortex and between the DN and the parietal cortex [24]. Subcortical networks such as the basal ganglia [7] are also connected with the cerebellum. These highlighted cerebellar cortical and subcortical networks are the anatomical clues for the role of the cerebellum in EES functions in adult humans.

Taken together, the reported findings suggest that the role of the cerebellum in EES function changes across the lifespan. In the following section, we propose that the cerebellum participates in a complex dynamic network that allows humans to acquire, develop and execute properly EES behaviors in real life. However, the significance of the cerebellum’s contribution to EES behavior is diminished in adults compared to children.

## The EES cerebellum in children

### Evidence of the role of the cerebellum in EES functions from healthy volunteer and patient studies

#### Studies in healthy volunteers

In healthy children, they are few task-based imaging studies related to EES functions [5, 42]. They are different from the adult studies (see above) in that, they do not only report activation clusters for a specific task, but they aimed to explore the relationship between cerebellar grey matter (GM) volume of a specific cerebellar region and performance of specific cognitive tasks. Moreover, the specific cerebellar regions explored are a priori chosen, based on the adult data. Therefore, the entire cerebellum is not explored for each task which limits the generalization of these results. Bernard et al. reported that larger GM volume of left Crus I and posterior cerebellum was correlated with lower working memory performance. This effect was not found for the right Crus I and posterior cerebellum [5]. Moore et al. reported an association between increased GM in bilateral lobule VIIB/VIIIA and left Crus II with higher scores on EF tasks as well as higher scores on a working memory task and GM in right lobule VII (Crus I/II). They noted that some GM activation clusters’ overlap between different cognitive tasks such as general intelligence ability [42]. Increased cerebellar GM volume and higher general intelligence ability were reported in children and young adults [17].

One study compared children and adult brain and cerebellar activation, using fMRI during a working memory task [9]. They reported that children and adults engaged different neural networks. Adults were engaging the prefrontal, the inferior temporal cortex, the posterior cingulate and the precuneus. Limited activation of the cerebellum was observed. On the other hand, children were engaging the premotor, superior/inferior parietal and middle temporal cortex, anterior insula, caudate/putamen, and the cerebellum. Cerebellar activations were stronger and more diffuse in children compared to adults [9]. Thus, there was a shift of the activation pattern, from children to adults, of areas closely related to sensory-motor and dorsal visual pathways associated with visual–spatial or action-related behaviors in children to complex cognitive processing in adults [13].

#### Studies in cerebellar patients

Since the latter part of the last century, cognitive deficits including EES functions were described following cerebellar injuries in children, leading to the launch of the Cerebellar Cognitive Affective Syndrome (CCAS) [35], and the Cerebellar Mutism Syndrome (CMS) [55]. In children, contrary to adults (see below), very few inconsistencies are reported [18].

The CCAS is characterized by impairments in EF (planning, set-shifting, abstract reasoning, verbal fluency, working memory), often with perseveration, distractibility or inattention; visual–spatial disorganization and impaired visual–spatial memory; personality change with blunting of affect or disinhibited and inappropriate behavior; and by impairments in language production such as dysprosody, agrammatism and mild anomia. The CMS is a condition that encompasses speech and language dysfunction (from complete mutism to mild dysfunction), behavioral/emotional changes with cerebellar motor signs (e.g., ataxia and gait disturbance), cognitive (including EF), and sometimes brainstem dysfunction including long tract signs and cranial nerve impairment. This syndrome has been extensively studied in children [55] especially after posterior fossa surgery but has also been described in a non-surgical context (cerebellitis) [38]. It has also been described in adults [56]. However, compared to children, different cerebellar sub-divisions were found to be responsible for CCAS onset (hemispheric lobules VI, VII and possibly lobule IX) with no involvement of the vermis [54].

The pathophysiology of CMS is unclear [4]. It occurs very rarely in adults [66]. However, in children, increasing evidence has shown an association between the CMS/CCAS and damage to cerebellothalamic-cerebral connections [3, 4, 56, 62] and to the deep cerebellar nuclei (DCN) [3, 32]. Also, bilateral DCN damage is likely to lead to greater deficits [3, 32]. Moreover, lesion mapping studies in children have found that damage to the vermis was associated with CMS/CCAS occurrence. Lobule IX and X of the vermis were identified as the sub-division responsible for the CMS/CCAS [3].

However, the impairment of a function after cerebellar damage may also reflect the remote effect of the cerebellar malfunction to the cerebral cortex. Perfusion Imaging studies are helpful to explore the distant consequence (s) of a cerebellar injury and therefore in understanding the role of the cerebellum in children [62]. In children with cerebellar pathologies, cerebellar perfusion deficits are reported [51, 65]. Supratentorial hypoperfusion was also reported which usually normalizes when cerebellar symptoms were resolved [41, 51, 65]. The hypoperfusion spreads to several cortical (e.g., frontal, parietal, temporal cortices) and subcortical (e.g., thalamus) regions with the frontal regions [from the PFC to the primary motor cortex (M1)] being the most consistently reported. One study found a global cortical hypoperfusion with a pronounced effect on the frontal lobes [41]. The mechanism of the distant cortical hypoperfusion is theoretically similar to the adult crossed cerebellar diaschisis initially described after a cortical stroke [8], however, for the cerebellum, cortical hypoperfusion is contralateral to the cerebellar affected hemisphere [41, 51, 65]. Furthermore, Wang et al. proposed the same diaschisis in ASD patients, in which disruption of the cerebello-thalamo-cortico pathways would lead to a developmental disorder leading to the appearance of ASD [64]. The diaschisis we refer here to is the connectional as opposed to the focal diaschisis in which changes of structural and functional connectivity between brain areas appear distant to the focal lesion. Connectional diaschisis explains the role of the cerebellum in the broad spectrum of high order functions due to its multiple and complex connections to distant cerebral cortical areas.

Up until recently, it was debatable whether a younger age at the time of a cerebellar injury predicted worse motor and cognitive outcomes. Does a cerebellar injury impair EES function in the same way across development? It has been shown that complex congenital cerebellar anomalies such as cerebellar agenesis may be linked to developmental diaschisis [64]. Others studies reported that cerebellar acquired damage at a young age contributed to more severe, and prolonged impairment [12, 50]. Some investigators argued that it was not due to the cerebellar injury but to the post-operative radiotherapy [44]. In non-irradiated children, no effect [27] or positive effect [35] of young age was reported. Only, Aarsen et al. described a negative effect [1]. However, multiple caveats limited those studies [6]. Beuriat et al. in a study of patients who were treated for a posterior fossa tumor, controlled all the major confounders (namely radiotherapy, tumor characteristics, damages to the deep cerebellar nuclei and delay between surgery and assessment time) and reported that early cerebellar damage (≤ 7 years old) is an independent factor for poor long-term functional (cognitive and motor) outcome [6].This suggests that the cerebellum is a sort of “broad learning machine”, and that during critical periods of development, the cerebellum, as part of a distributed neural network, is the foundation for future motor and cognitive learning.

This concept has been previously suggested by some authors. In a consensus paper on the role of the cerebellum in movement and cognition, most of the authors described the cerebellum as a structure that acquires internal models that govern both movement and thinking [29]. Schmahmann defined it as a Universal Cerebellar Transformation [53] while Riva and colleagues characterized the cerebellum as a “homeo-static orchestrator” [49] and Parker and Andreasen's described the concept of “synchrony” as the coordination of sequences of both action (movement) and thought [45]. This is also consistent with the view that the cerebellum is critical to motor and cognitive automation and adaptation as supported by others [43]. It was also proposed that the cerebellum operates as a general-purpose co-processor, whose effects depend on the specific brain centers to which individual modules are connected [11]. The idea of these authors is that the cerebellum act as a “timing and learning machine” for cognitive/emotional and mental processes.

### Cerebellar anatomy and EES networks in children

Structurally, the cerebellum and the cerebrum undergo major changes during human development, as a consequence of brain maturation and adaptation. Functionally, networks evolve from spatially close anatomical hubs to large-scale but integrated network communities [15].

#### Structural modifications

Most of the brain’s macroscopic changes (cortical folding and increase volume) are completed by the age of 2 [46]. Nevertheless, the course of maturation of the cortex is different from one region to another with a later maturation of cortices processing high-order functions compared to those processing somatosensory ones. For example, somatosensory, visual, auditory, and motor cortices mature earlier than the PFC. One the other hand, microstructural (sub-cortical areas/white matter of the brain and cerebellum) architecture continues to mature from gestational age to far beyond 2 years of age. These changes are supported by the myelinization process that continues into young adulthood [19]. EES and complex sensorimotor behavioral development are more limited before 2 years old with later development [46] including myelinization, leading to an increased efficiency of the connectivity of these large-scale integrated networks [16].

Cerebellar macroscopic changes reaches peak later in development [61]. Interestingly, the cerebellar vermis, has an almost stable volume from 8 to 20 years old. Most of the later growth of the cerebellum is due to the increased volume of the cerebellar hemispheres [61].

#### Brain and cerebellar functional connectivity and EES network modifications

Using resting-state functional magnetic resonance image (rs-fMRI), it was reported that functional connectivity (FC) is already present in utero from 24 weeks gestation age and increases with full-term age [60]. However, this connectivity between brain regions is immature (weak FC) compared to adults, even at full term, especially antero-posterior networks compared to inter-hemispheric ones [60]. Using diffusion magnetic resonance image (dMRI), Huang et al. showed that the number of connections (nodes) within the functional networks was lower in neonates compared to toddlers and preadolescents [23].

**Fig. 3:**
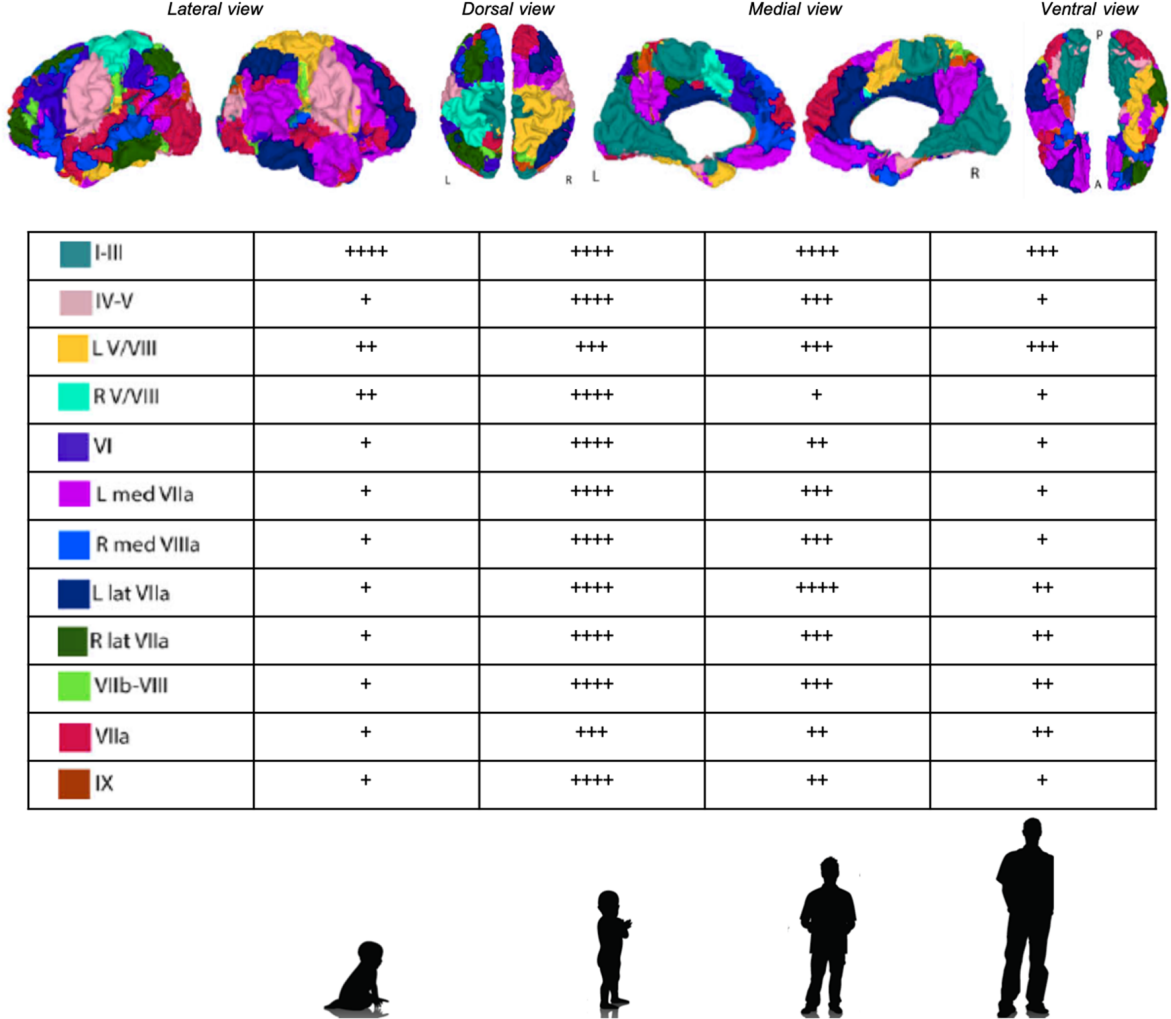
Cerebello-cortical functional networks from early childhood to adulthood: this figure illustrates the cerebello-cortical functional networks according to the functional connectivity of the cerebellar networks as proposed by Yeo et al. [68]. For each cerebellar lobules, the cerebello-cortical connectivity strengh at each age (early childhood, middle childhood, late childhood, adulthood) is represented from low (+) to high (++++). Note that the maturity of the cerebello-cortical functional networks dysplayed here increases from early childhood to adulthood. Maps are overlaid on anatomical surface maps. *A* anterior; *P* posterior; *L* left; *R* right; *lat* lateral; *med* medial

It had been thought that long-range network connections between the cerebellum and cortical areas developed only after birth [67]. Yet, the cortical volume of prefrontal, sensorimotor and temporal regions is decreased after preterm cerebellar injury [37]. Contrasting results were published regarding the FC of the cerebellum with other cortical regions in children. Doria et al. found FC between the cerebellum and cortical regions such as the PFC, cingulum and parietal lobe in full term infants at birth. The connectivity in this network was not present in the early preterm infant [14]. In contrast, Kipping et al. showed that in the infant (6 months of age), the cerebellum connection to the sensorimotor cortex was present, but that connections to other cortical regions only appeared later in childhood. In his study, the sensorimotor cortex was connected to lobule I–IV and to lobule VIII. This is remarkable because, in the adult humans, lobule VIII is said be part of the cognitive part of the cerebellum. The strength of cortico-cerebellar FC was greater in middle childhood compared to infant or late childhood and even to the adult. The coherence (maturity), of the cortico-cortical FC was stronger in adults. Therefore, they described the development of Cerebellar, Cerebello-Cortical, and Cortico-Cortical Functional Networks in Infancy, Childhood, and Adulthood as “asynchronous” [25] (Fig. 3).

Moreover, Kipping et al. assessed cerebellar GM volume and white matter (WM) tract integrity [Fractional Anisotropy (FA)] in children from 6 to 10 years of age. First, they showed that there was an evolution across development of both parameters. Compared to older children, younger children had lower GM volumes and higher WM FA of the anterior cerebellum, while compared to younger children, older children had higher GM volumes and lower WM FA in the posterior cerebellum. Moreover, they correlated these findings with increasing cognitive planning capacity [26].

The evidence reviewed above indicates significant morphological, microstructural and functional reorganization of human cortical and cerebellar structural networks from infancy to adulthood. As this occurred, EES functional processing became more efficient and organized indicating that the relevance of each component of the network to EES changes over development. We will address later in this article the implication of these changes for the shifting role of the cerebellum in EES functions.

## The shifting cerebellar role in ees processing from childhood to adulthood

Having identified the cerebellum as an important anatomical hub does not mean that its role in a network is necessarily crucial. Indeed, every functional network is built on regional nodes some of which can be considered crucial to the network’s assigned functional role(s) whereas other network nodes can be considered supportive. For example, even if the primary visual cortex (V1) is a member of an extended network that reaches into the prefrontal cortex, it would be hard to argue that V1 has a crucial role in decision-making or social cognition. In this example, V1 may provide visual information contributing to a decision or social interaction but is not likely to be computing the higher-order representations that are crucial to the decision (e.g., social context, procedures, and rules). Likewise, higher-order decision making can bias how we process visual information (e.g., by directing visual attention to a particular object’s orientation) but is not likely to be computing the molecular aspects of visual information processing.

### The crucial to supportive evolution of the cerebellum’s role in EES functioning

Movement and cognition are human behaviors that are deeply intermingled in our daily life. In newborns, both motor and cognitive behaviors are relatively “primitive”. In late childhood, our motor abilities are approaching their peak of performance, but our cognitive behaviors continue to mature—especially EES-related behaviors—into young adulthood. We propose that EES-related behaviors are more dependent upon motor processing in newborn and younger children given their cognitive immaturity.

Clinical studies reporting the outcome of patients with acute cerebellar injury or chronic neurodegeneration have shown that the degree of cognitive impairment and the long-term effect of the injury is worse in younger children [1, 6]. Preterm cerebellar injuries are also associated with poor motor and cognitive outcome [59]. Moreover, other studies reported an association between motor and executive function impairment in children [28].

When damaged, inflow and outflow pathways to/from the cerebellum, especially via the thalamus, not only play a role in both ataxic movement and impaired fine motor skills, but also EES dysfunction, particularly after childhood cerebellar injury [3]. This is because the cerebellum is acting more “independently” from the cerebrum in newborns and young children with less efficient feedback and feedforward capacities. But, since it may mature earlier than many other nodes in the network, such as the PFC, that builds motor and high-order cognitive behaviors influencing EES functions, the cerebellum’s role is more important to the perception and execution of EES functions in infants and young children than many other nodes. That means that children’s EES behaviors are more dependent upon observing the timing and sequence of EES-related actions than the conceptual basis of those same actions.

Two other observations support this hypothesis. The first one is that, in children, the PFC is not increasingly recruited when performing an EF task when the cerebellum is damaged in contrast to what is observed in adults [31]. In an immature, less well-connected and not fully functional network, damage to one of the nodes in the network cannot be easily compensated for by other nodes. The second observation stems from the difference between the adult crossed cerebellar diaschisis and the childhood CCAS/CMS cortico-cerebellar diaschisis. In adults, a crossed cerebellar diaschisis after a cortical injury such as a stroke due to cerebellar contralateral hypoperfusion may be observed. In this case, no additional clinical symptoms, due to the hypoperfused cerebellum, have been identified in humans, non-human primates or rodents [36]. On the contrary, in children, EES functional impairments may be due to the secondary remote cortical hypoperfusion [41, 51, 65]. This indicates that in an adult with a mature, well-connected and fully functional network, hypoperfusion of the cerebellum has much less impact because other nodes in the network are sufficient to compensate for its diminished functionality. If the cerebellum was a crucial node in the EES network in the adult and was damaged, chronic EES deficits should be apparent not only immediately after the damage but also when the patient has reached a plateau in recovery.

## Conclusions

For every function, different cortical and sub-cortical regional nodes can be considered crucial to any network they belong to or the nodes can simply be supportive, supplying information for, or biasing, decision-making and agency. To complicate matters, the relative importance of cerebellar participation in the network(s) evolves during development. This evolution is driven by the macroscopic and microscopic modifications of the cerebellum that are occurring during development including its increasing connectivity with distant supra-tentorial cortical and sub-cortical regions.

**Fig. 4:**
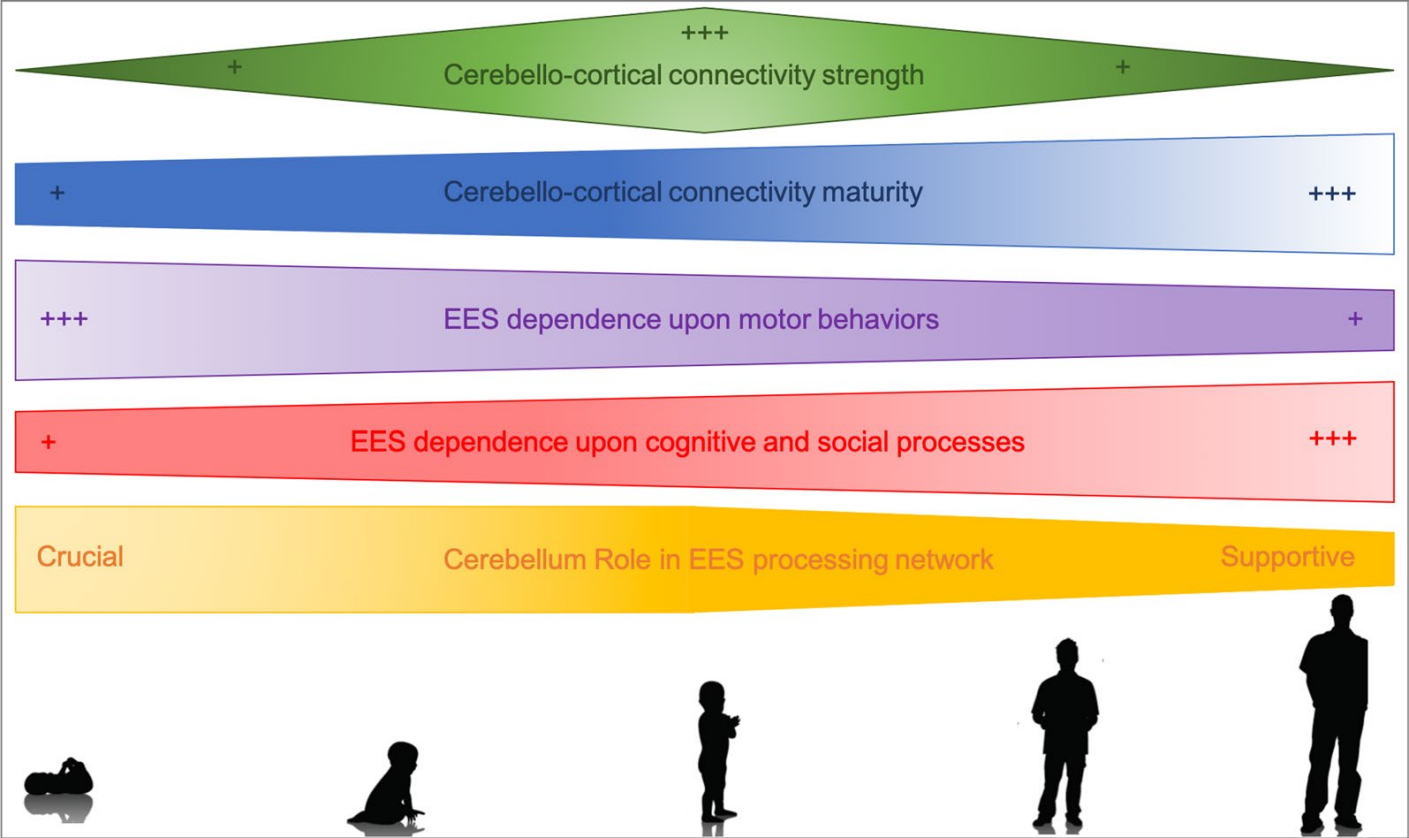
Summary of the asynchronous development of EES functions and Cerebello-Cortical Networks from Infancy to Adulthood that supports the shifting role of the cerebellum in EES processing network. For each item the shape of figure represents the evolution of its relative importance across development. *+* weak/low; *+++* strong/high

As a result of these anatomical and functional changes, neuroimaging and clinical data indicate that the importance of the role of the cerebellum in human EES-related networks shitfs from being crucial in newborns and young children to being only supportive later in life. In early life, given the immaturity of cortically mediated EES functions, EES functions and motor control and perception are more closely interrelated. At that time, the cerebellum due to its important role in motor control and sequencing makes EES functions more reliant on these computational properties that compute spatial distance, motor intent, and assist in the execution of sequences of behavior related to their developing EES expression. As the cortical brain matures, EES functions and decisions become less dependent upon these aspects of motor behavior and more dependent upon high-order cognitive and social conceptual processes. At that time, the cerebellum assumes a supportive role in these EES-related behaviors by computing their motor and sequential features (Fig. 4). We suspect that this evolving role of the cerebellum has complicated the interpretation of its contribution to EES computational demands.

However, in order to truly understand this shift in nodal network responsibility, future longitudinal cohort studies are needed that follow healthy participants from in utero to later in adulthood, using repeated functional and morphological imaging and neuropsychological testing to monitor the developmental relationship between cerebellar structure and cognition including EES functions. Moreover, longitudinal cohort studies in cerebellar patients are also needed to compare long term sequelae across all age but also to evaluate the pattern of recovery depending on age.

## Data Availability

Not applicable.
